# EXPLORING THE IMPACT OF NURSE WAGES ON NURSING HOME QUALITY

**DOI:** 10.1093/geroni/igae098.0534

**Published:** 2024-12-31

**Authors:** Rohit Pradhan, Akbar Ghiasi, Gregory Orewa, Ganisher Davlyatov, Robert Weech-Maldonado

**Affiliations:** Texas State University, San Marcos, Texas, United States; University of the Incarnate Word, San Antonio, Texas, United States; The University of Texas San Antonio, San Antonio, Texas, United States; University of Oklahoma Health Sciences Center, Oklahoma City, Oklahoma, United States; University of Alabama at Birmingham, Birmingham, Alabama, United States

## Abstract

Nurses are the primary caregivers in nursing homes (NHs) and providing high-quality care hinges significantly upon their adequacy and expertise. However, NHs have historically struggled with inadequate nurse staffing, partly due to nurse wages in NHs being lower than those in other healthcare settings, such as hospitals. This study aimed to explore the relationship between nurse wages and the quality of care in NHs. This study used secondary datasets such as the Payroll-Based Journals and Care Compare (2017-2022). Multivariate ordinal logistic regression with two-way (state and year-level) fixed effects was employed. The study included all U.S. NHs certified by the Centers for Medicare and Medicaid Services (CMS). Analytic data comprised 80,244 facilities, averaging 13,374 unique NHs per year. We focused on the CMS NH Five-Star Quality Rating System’s quality star rating (1-5 scale) as the dependent variable, with independent variables being the inflation-adjusted hourly wages for registered nurses (RNs), licensed practical nurses (LPNs), and certified nursing assistants (CNAs), while controlling for facility and community characteristics that influence quality. The findings revealed that a one-dollar increase in hourly RN wages was linked to a three percentage-point increase in the odds of an NH receiving a higher star rating (p<.01). Increased RN and CNA hours per resident day (PRD) were positively associated with higher quality ratings, whereas higher LPN hours PRD were correlated with lower quality ratings (p<.001). These results highlight the need for targeted policy and managerial interventions to adjust nurse compensation strategies, particularly for RNs, to enhance NH quality.

